# The Effect of Oligopin Supplementation on Hormonal and Metabolic Profiles in the Polycystic Ovary Syndrome: A Randomized Controlled Trial

**DOI:** 10.3389/fendo.2020.590392

**Published:** 2020-12-21

**Authors:** Mostafa Qorbani, Milad Sanginabadi, Mohammad Reza Mohajeri-Tehrani, Sara Karimi, Hadis Gerami, Armita Mahdavi-Gorabi, Nooshin Shirzad, Majid Samadi, Fereshteh Baygi, Saeed Hosseini, Asieh Mansour

**Affiliations:** ^1^ Non-Communicable Diseases Research Center, Alborz University of Medical Sciences, Karaj, Iran; ^2^ Chronic Diseases Research Center, Endocrinology and Metabolism Population Sciences Institute, Endocrinology and Metabolism Research Institute, Tehran University of Medical Sciences, Tehran, Iran; ^3^ Radiology Department, Shariati Hospital, Tehran University of Medical Sciences, Tehran, Iran; ^4^ Endocrinology and Metabolism Research Center, Endocrinology and Metabolism Clinical Sciences Institute, Tehran University of Medical Sciences, Tehran, Iran; ^5^ Department of Clinical Nutrition and Dietetics, Faculty of Nutrition and Food Technology, National Nutrition and Food Technology Research Institute, Shahid Beheshti University of Medical Science, Tehran, Iran; ^6^ Social Determinants of Health Research Center, Alborz University of Medical Sciences, Karaj, Iran; ^7^ Department of Endocrinology, Vali-Asr Hospital, Endocrinology and Metabolism Research Center, Imam Khomeini Complex Hospital, Tehran University of Medical Sciences, Tehran, Iran; ^8^ Centre of Maritime Health and Society, Department of Public Health, University of Southern Denmark, Esbjerg, Denmark; ^9^ Department of Clinical Nutrition, School of Nutritional Sciences and Dietetics, Tehran University of Medical Sciences, Tehran, Iran

**Keywords:** oligopin, polycystic ovary syndrome, PCOS, endocrine, metabolic profile

## Abstract

**Background:**

A double blind clinical trial was performed to evaluate whether the polycystic ovary syndrome (PCOS)-specific serum markers and metabolic parameters would change in the women with PCOS during the three-month administration of oligopin.

**Methods:**

In this double-blind multicenter trial, we randomly assigned 80 PCOS women, based on a 1:1 ratio, to receive oligopin (n= 40) or maltodextrin as placebo (n = 40) for up to 3 months. As PCOS-specific outcomes, we investigated the changes in testosterone, sex hormone binding globulin (SHBG), free androgen index (FAI), dehydroepiandrosterone (DHEA), *follicle-stimulating* hormone (*FSH*) and *luteinizing hormone* (*LH*). Secondary end points were metabolic (fasting glycaemia, hemoglobin A1c (HbA1c), lipids, insulin resistance (HOMA-IR)), anthropometrics parameters and blood pressure from the baseline to the end of treatment. We investigated serum transaminase, alkaline phosphatase (ALP), creatinine (Cr) and blood urea nitrogen (BUN) levels as hepatic and kidney outcomes, respectively.

**Results:**

The first participant was enrolled on April 18, 2018, and the last study visit took place on May 14, 2019. PCOS-specific serum parameters did not change during the three-month administration of oligopin (p > 0.05), except for a small increase in the FSH levels (p=0.03). Oligopin neither changed the metabolic profile nor the anthropometric parameters or blood pressure. ALP levels was significantly increased in placebo group, as compared with oligopin (p=0.01).

**Conclusion:**

Oligopin supplementation does not seem to be exerting a beneficial effect on both hormonal and metabolic parameters in the women with PCOS.

**Clinical Trial Registration:**

www.irct.ir, identifier IRCT20140406017139N3.

## Background

*Polycystic ovary syndrome* (PCOS) is the most common endocrine-metabolic disorder causing the infertility, affecting 5%–20% of women in their reproductive lifespan (1). PCOS can often be characterized by elevated circulating androgen levels, hirsutism, acne, oligomenorrhea or amenorrhea, and/or polycystic ovarian morphology (PCOM), as determined by ultrasound. Endocrine and metabolic derangements and cardiovascular disorders may also coexist (2). Hyperinsulinemia, a consequence of insulin resistance, can cause hyperandrogenism, leading to the inappropriate gonadotropin secretion (reduction of *follicle-stimulating* hormone (*FSH*) and the increase of *luteinizing hormone* (*LH*) levels) in *PCOS* (3).

Oxidative stress has been implicated in mediating the insulin resistance and excessive ovarian androgen levels seen in these patients (4, 5). Based on the large evidence showing the relationship between oxidative stress and impaired insulin action (6) and hyperandrogenism, an independent correlation between antioxidant decline and PCOS has been reported (7). In the recent years, several studies have demonstrated the efficacy of antioxidants such as bioflovonids in reducing PCOS-associated hyperinsulimemia and correcting common endocrine and metabolic dysfunctions found in the women with PCOS (8). A lower circulation level of LH/FSH has been reported in isoflavone consumption, as compared to the controls in the meta analysis (9). Moreover, flavonoids, as a potent antioxidant, have been demonstrated to reduce the clinical and biochemical markers of hyperandrogenism in the PCOS patients (10), as well as in the animal models affected by PCOS (11, 12). Such findings have encouraged the development of other flavonoids-rich medicinal plants for the treatment of PCOS

Among the available compounds, oligopin (a pine bark extract of the French maritime pine), a plant extract containing procyanidins (catechin and epicatechin), protects tissues from oxidative stress and the inflammation-related damage due to its strong antioxidant and anti-inflammatory activity (13, 14). Furthermore, animal studies have demonstrated that the pine bark extract and derivatives may affect the ROS levels and lipid accumulation by the inhibition of ROS production through the mechanism associated with pro -oxidant and antioxidant enzyme responses and the suppression of the adipogenic gene expression in adipocytes (15). Administration of the pine bark extract was also shown to reduce fasting glucose levels and HbA1c levels, as compared to the control, through several mechanisms (16, 17). Pycnogenol may stimulate the glucose uptake *via* the *PI3K*/*AKT* signaling *pathway* (18). In addition, it is suggested that the reduced blood glucose, by the inhibition of α-glucosidase activity, may reduce glucose absorption in the intestinal cells (19). A very recent meta -analysis aimed to investigate the effect of pycnogenol on the cardiometabolic factors in healthy and affected individuals (16); notably, there has been no study assessing the effects of this extract on the PCOS women. Therefore, the aim of the present study was to assess the efficacy and safety of the 3-month oligopin administration on the hormonal and metabolic features of the women affected by PCOS.

## Methods

### Study Design

This trial (IRCT.IR identifier) was a 3-month randomized placebo-controlled double blind trial performed at three university hospitals in Tehran, Iran. Patients were included if they were in the age range of 18-40 years and provided their written informed consent. Further, PCOS was documented according to the Rotterdam criteria if two out of the three following conditions were met: a) oligomenorrhea (menstrual cycle>35 days) or amenorrhea, b) clinical and/or biochemical signs of hyperandrogenism, and c) polycystic ovaries (≥12 follicles of 2–9 mm diameter on at least one ovary and/ or *ovarian volume* ≥*10* ml) on the abdominal ultrasound (20). Key exclusion criteria included pregnancy, history of diseases that could cause menstrual disturbances (e.g., elevated prolactin and thyroid disease), or the use of drugs known to influence metabolism (like metformin or *glucocorticoid)* and ovarian functions (such as oral contraceptives) for at least 30 days or more before screening; also, evidence of diabetes, significant liver and renal impairment, Cushing’s syndrome and acromegaly were the other exclusion criteria.

Ethics approval was obtained from the Ethics Committee of Endocrinology Metabolism Research Institute, Tehran University of Medical Sciences (REC.1396.00163). This study adhered to the CONSORT guidelines.

### Randomization

Patients were randomized, based on a 1:1 ratio, according to the method of block randomization; sample size was estimated 40 patients per groups using two mean comparison sample size formula, with power 80% and test of significance at 5% level (a standard deviation of 24 mIU /L for the FSH of either group was assumed) (21). Placebo was provided in identical intakes; the recommended intake was oligopin 50 mg/day or placebo (maltodextrin), for the whole duration of the study (3 months).

### Procedures

The trial was undertaken in the early follicular phase (cycle days 3–7) in regularly menstruating women or random days for women with oligo/amenorrhea. The height was measured with a wall-mounted meter with an approximation of 0.5 cm. Body weight and composition were measured without footwear and with light clothing, using the body impedance analyzer (BIA) (Tanita, Japan). The circumferences of waist were measured as the value between the iliac crest and the lateral costal margin. Body mass index (BMI) = weight (kg)/ height (m^2^) was calculated as well. The grade of hirsutism was established using the Ferriman-Gallwey score (22). Acne was evaluated in four grades, as described previously (23).

A trans-abdominal ultrasound was performed by one of two well-trained radiologists using a 3 MHz to 5.5 MHz curvilinear probe (acuson s2000, Siemens Medical Solutions, USA). Ovarian volume was calculated for each ovary using the prolate ellipsoid formula: π/6 *maximum diameter in transverse*anteroposterior * longitudinal axes (24). The total number of antral follicles (2–10 mm in diameter) was counted (25).

The blood samples were collected from 8:00 to 9:00 AM after following overnight fasting, frozen and stored at 20°C until analysis. The serum levels of sex hormone binding globulin (SHBG) were assessed using ELISA kits (Demeditec, Germany). The free androgen index (FAI) was calculated (FAI) = testosterone (ng/ml) × 3.47/SHBG (nmol/L) (26). All remnant hormonal (dehydroepiandrosterone (DHEA), testosterone, FSH, LH, prolactin, C-peptide, insulin and thyroid stimulating hormone (TSH)) assays were performed with the ELISA kits (Monobind Inc. Lake Forest, California, USA). Fasting blood glucose levels were measured using the glucose oxidase method on an autoanalyzer (Cobas c 311, Roche Dignostics, Risch-Rotkreuz, Switzerland). Hemoglobin A1c (HbA1c) level was determined on a daily basis using a high performance liquid chromatography analyzer (Tosoh, Tokyo, Japan). Low-density lipoprotein cholesterol (LDL) was calculated using the Friedewald formula. The insulin resistance was calculated based on the Homeostasis Model Assessment (HOMA): HOMA-IR= (fasting glucose (mg/dl)) × (fasting insulin (µIu/ml))/405 (27). Concentrations of the total cholesterol (TC), triglycerides (TG), high-density lipoprotein cholesterol (HDL), liver function tests (alanine transaminase (ALT), aspartate transaminase (AST) and Alkaline phosphatase (ALP)), kidney function tests ( blood urea nitrogen (BUN), creatinine (Cr)) and high sensitive- C reactive protein (hs-CRP) were measured by applying the ELISA kit (Roche, Germany).

### Statistical Analysis

Statistical analysis was carried out using the SPSS, version 16.0 (SPSS Inc., Chicago, IL, USA) on an intention-to-treat basis. Continuous variables were evaluated for normality by applying the Shapiro-Wilk test. Non-normally distributed variables were transformed using an appropriate transformation method. Continuous and categorical variables were reported as the mean [standard deviation (SD)] and number (%), respectively. Comparison of the continuous variables in the oligopin and placebo groups at the baseline was done using independent-samples t-tests. Two-way repeated-measures of ANOVA were also used to assess the effect of intervention on the continuous outcomes. Chi-squared test was also employed to assess the categorical variables at the baseline in the groups. All statistical tests were two-tailed; P< 0.05 was considered as the threshold significant level.

## Results

**Figure 1:**
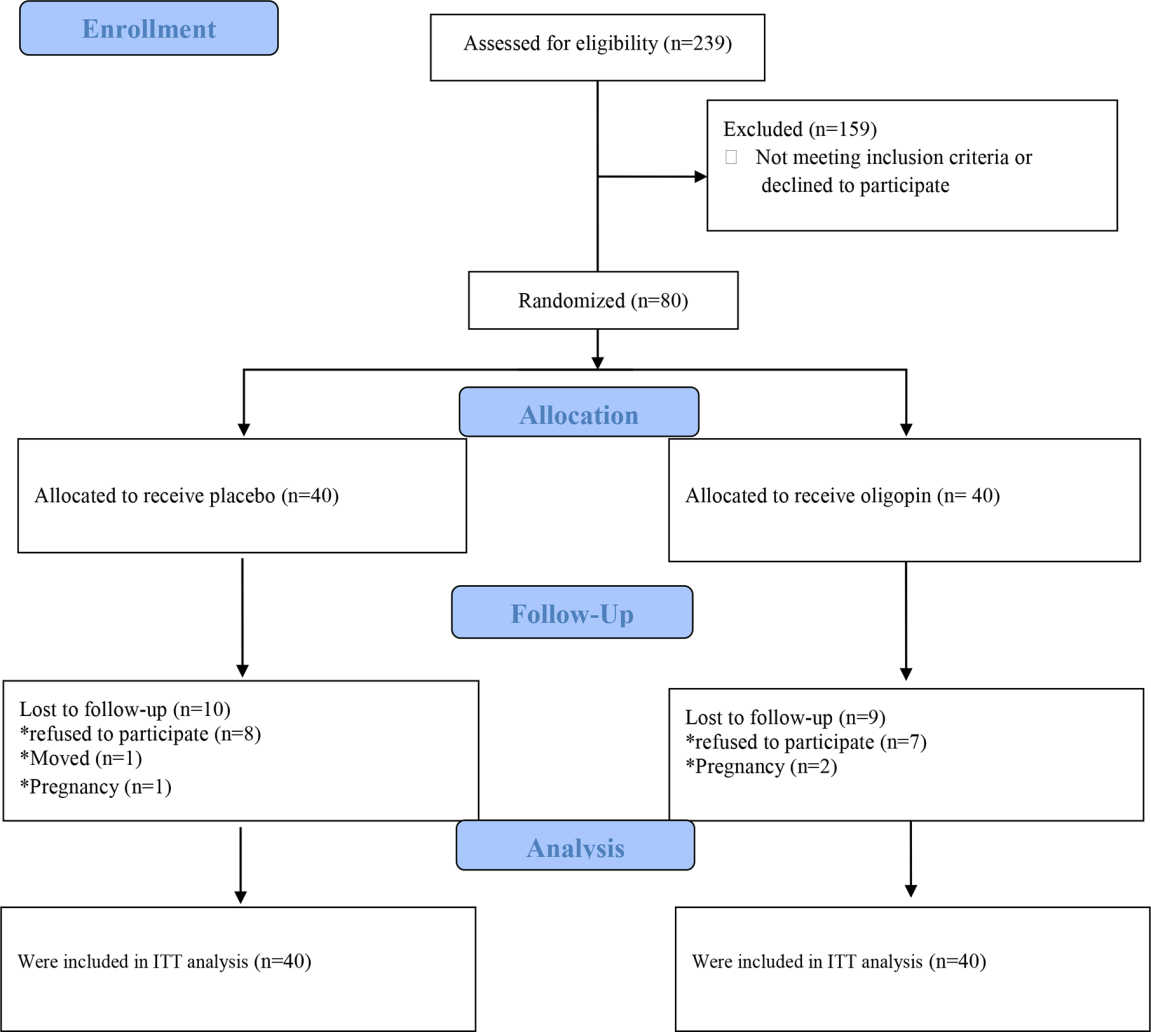
Trial profile.

The first participant was enrolled on April 18, 2018, while the last patient visit was on May 14, 2019. Of 239 patients assessed for eligibility, 80 were enrolled and randomly assigned to treatment with once-daily 50 mg oligopin (n=40) or placebo (n=40) (**Figure 1**). The treatment schedule was completed by 31(77.5%) participants in the oligopin group and 30 (75%) in the placebo group. Baseline characteristics are shown in **Table 1**. The mean (SD) age was 27.99 ± (6.28) years, the mean disease duration was 6.17 years, and the majority were non-smokers (98.8%); they had irregular menses (80%). Hirsutisim degree was different in the two groups at the baseline (p=0.008) (**Table 1**). As the safety, no serious oligopin-related adverse events occurred during the study.

**Table 1:** Baseline characteristics of patients according to study group.

	Placebo (n= 40)	Oligopin (n=40)	P value
Age (year)	27.6±6	28.38±6.57	0.58^a^
Smoking (yes) n(%)	0	1(2.5)	0.31^b^
Height(meters)	160.05±5.91	159.72±5.67	0.8^a^
Disease duration (year)	4.83±5.69	7.51±7.28	0.07^a^
Menes n(%)			
Regular irregular	8(20) 32(80)	8(20) 32(80)	0.99^b^
Hair losses (yes) n(%)	22(64.7)	32(80)	0.14^b^
Hirsutism	17.28±5.54	21±6.58	0.008^a^
Acne score	1.5±1.48	1.55±1.39	0.87^a^

a Independent samples t-test.

b chi-square.

Each value represents mean± SD except for smoking n(%), menes n(%), hair losses n(%).

Regarding the primary endpoints, changes in the androgen levels such as testosterone, SHBG, FAI and DHEA levels, from the baseline to 3 months, did not differ significantly in the two study groups (p>0.05), except for the increase of the FSH levels in the oligopin group (the mean difference of 0.62 mIU/ml [95%CI, 0.04 to 1.19]), as compared with the placebo ( the mean difference of −0.41 mIU/ml [95%CI, −1.13 to −0.33]), p=0.03 (**Table 2**). Similarly, none of the indicators of metabolic control (fasting blood glucose, HbA1c, insulin levels, lipid profile), hs-CRP levels and anthropometric parameters [BMI, fat free mass (FFM), fat mass (FM), waist circumference] showed any significant changes in the oligopin and placebo groups after 3 months (**Tables 2**, **3**). Changes in ALP at the end of the trial differed in the oligopin (the mean difference of 4.66 U/L [95%CI, −1.14 to 10.45]) and placebo groups (the mean difference of 25.15 U/L [95%CI, 11.08 to 39.21]), (p=.0.01). However, changes in other transaminases levels (ALT and AST) and kidney function factors (Cr, BUN) and TSH levels were not significantly different in treatment and placebo groups (P>0.05) (**Table 2**). Parameters related to blood pressure (systolic and diastolic) were also similar in the PCOS patients after oligopin treatment (**Table 3**).

**Table 2:** Hormonal and metabolic parameters at baseline and after 3 months of treatment with oligopin and placebo in the study population.

	Placebo (n=40)	Oligopin (n=40)	P value
	means (95%CI)	means (95%CI)	
Testosterone (ng/ml)			
Baseline	0.41(0.34 to 0.48)	0.44(0.38 to 0.51)	
12 weeks	0.41(0.34 to 0.50)	0.46(0.38 to 0.55)	0.84^a^
DHEA (ng/dl)			
Baseline	160.02(138.53 to 183.06)	155.07(133.81 to 177.87)	
12 weeks	131.14(106.91 to 157.65)	151.16(125.19 to 179.56)	0.13^a^
SHBG (nmol/l)			
Baseline	44.77(35.16 to 57.02)	33.11(26 to 42.17)	
12 weeks	40.83(33.8 to 49.2)	31.77(26.3 to38.28)	0.74^a^
FAI			
Baseline	2.98(2.20 to 4.03)	4.33(3.20 to 5.87)	
12 weeks	3.19(2.47 to 4.11)	4.84(3.75 to 6.24)	0.84^a^
LH (mIU/ml)			
Baseline	10.44(7.57 to 14.42)	8.95(6.48 to 12.33)	
12 weeks	9.88(7.83 to 12.47)	10.66(8.45 to 13.45)	0.34^a^
FSH (mIU/ml)			
Baseline	5.76 (5.13 to 6.4)	5.16(4.52 to 5.8)	
12 weeks	5.35(4.91 to 5.79)	5.79(5.35 to 6.23)	0.03^a^
LH/FSH			
Baseline	1.96(1.51 to 2.54)	1.88(1.45 to 2.43)	
12 weeks	1.9(1.52 to 2.38)	1.91(1.52 to 2.38)	0.8^a^
Prolactin (ng/ml)			
Baseline	14.69(12.79 to 16.86)	13.49(11.75 to 15.50)	
12 weeks	15.27(13.40 to17.42)	14.38(12.62 to 16.40)	0.73^a^
TSH (mIU/ml)			
Baseline	2.28(1.95 to 2.63)	1.98(1.67 to 2.31)	
12 weeks	2.69(2.34 to 3.1)	2(1.69 to 2.34)	0.15^a^
Insulin (µIU/ml)			
Baseline	12.98(11.41 to 14.55)	13(11.43 to 14.57)	
12 weeks	13.41(11.95 to 14.86)	12.31 (10.85 to 13.76)	0.41
C-peptide (ng/ml)			
Baseline	1.17(1 to 1.35)	1.32(1.15 to 1.5)	
12 weeks	1.25(1.12 to 1.38)	1.32(1.19 to 1.46)	0.44^a^
Fasting blood sugar (mg/dl)			
Baseline	88.3(85.29 to 91.31)	89.32(86.32 to 92.33)	
12 weeks	88.62(86.27 to 90.97)	90.486(88.14 to 92.83)	0.69^a^
HOMA-IR			
Baseline	2.58(2.22 to 3)	2.63(2.26 to 3.06)	
12 weeks	2.73(2.42 to 3.1)	2.58 (2.28 to 2.92)	0.55^a^
HbA1C			
Baseline	5.24(5.14 to 5.33)	5.29(5.19 to 5.39)	
12 weeks	4.82(4.6 to 5.05)	4.93(4.7 to 5.16)	0.76^a^
Triglyceride (mg/dl)			
Baseline	98.17(86.09 to 112.20)	109.14(95.5 to 124.45)	
12 weeks	98.17(85.9 0to 111.94)	105.20(92.04 to 119.94)	0.56^a^
Cholesterol (mg/dl)			
Baseline	168.72(158.24 to 179.20)	177.45(166.97 to 187.93)	
12 weeks	165.55(155.73 to 175.37)	174(164.18 to 183.82)	0.94^a^
HDL-C (mg/dl)			
Baseline	41.57(38.6 to 44.54)	46.27(43.3 to 49.24)	
12 weeks	42.03(39.35 to 44.7)	46.09(43.41 to 48.75)	0.65^a^
LDL-C (mg/dl)			
Baseline	96.38(89.33 to 103.99)	98.85(91.83 to 106.65)	
12 weeks	95.49(88.92 to 102.32)	98.85(92.26 to 105.92)	0.76^a^
AST (U/L)			
Baseline	19.01(17.74 to 20.41)	18.7(17.39 to 20.23)	
12 weeks	16.18(15.06 to 17.33)	16.14(14.92 to 17.45)	0.7^a^
ALT (U/L)			
Baseline	10.52(9.43 to 10.53)	9.61(8.69 to 10.75)	
12 weeks	10.64(9.26 to 12.5)	8.47(7.57to 9.61)	0.21^a^
ALP (U/L)			
Baseline	122.37(104.77 to 139.97)	149.52(131.93 to 167.12)	
12 weeks	147.52(129.32 to 165.74)	154.19(135.98 to 172.39)	0.01^a^
hs-CRP (mg/L)			
Baseline	1.03(0.72 to 1.47)	1.61(1.12 to 2.3)	
12 weeks	1.32(0.92 to 1.89)	1.77(1.24 to 2.54)	0.38^a^
Urea (mg/dl)			
Baseline	22.37(20.48 to 24.27)	22.97(21.08 to 24.86)	
12 weeks	23.35(21.25 to 25.43)	23.57(21.48 to 25.66)	0.75^a^
Creatinine (mg/dl)			
Baseline	0.78(0.75 to 0.82)	0.86 (0.82 to 0.89)	
12 weeks	0.8(0.77 to 0.84)	0.87(0.83 to 0.91)	0.63^a^

a Time to treatment interaction according to two-way repeated measure ANOVA. Data are not conforming to a normal distribution were log or square root or inverse transformed.

DHEA, dehydroepiandrosterone; SHBG, sex hormone-binding globulin; AFI, free androgen index; LH, luteinizing hormone; FSH, follicle-stimulating hormone; TSH, thyroid stimulating hormone; HOMA-IR, homeostasis model assessment insulin resistance; HbA1c, hemoglobinA1c; HDL-C, high-density lipoprotein cholesterol; LDL-C, low-density lipoprotein cholesterol; AST, aspartate aminotransferase; ALT, alanine aminotransferase; ALP, alkaline phosphatase; hs-CRP, high-sensitivity C-reactive protein.

**Table 3:** Anthropometrics, blood pressure and physical activity at baseline and after 3 months of treatment with oligopin and placebo in the study population.

	Placebo (n=40)	Oligopin (n=40)	P value
	means (95%CI)	means (95%CI)	
Body mass index (kg/m^2^)			
Baseline	26.3(24.55 to 27.54)	27.54(25.7 to 29.51)	
12 weeks	26.3(24.54 to 28.18)	28.18(26.66 to 30.06)	0.5^a^
Fat mass(kg)			
Baseline	22.85(20.09 to 26)	27.41(24.10 to 31.19)	
12 weeks	23.5(20.84 to 26.48)	28.38(25.18 to 31.99)	0.87^a^
Fat free mass (kg)			
Baseline	43.35(41.97 to 44.67)	42.36(41.02 to 43.65)	
12 weeks	43.35(42.17 to 44.46)	43.05(41.88 to 44.15)	0.14^a^
Waist circumference(cm)			
Baseline	91.62(87.7 to 95.94)	93.32(89.12 to 97.5)	
12 weeks	92.47(88.71 to 96.38)	93.32(89.53 to 97.27)	0.59^a^
Systolic blood pressure (mmHg)			
Baseline	111.15(106.34 to 115.96)	104.3(99.54 to 109.05)	
12 weeks	107.36(102.56 to 112.16)	101.83(97.09 to 106.57)	0.67^a^
Diastolic blood pressure (mmHg)			
Baseline	76.28(72.54 to 80.02)	73.25(69 to 76.95)	
12 weeks	73.99(70.44 to 77.54)	69.46(65.95 to 72.96)	0.49^a^
Physical activity (METs/h)			
Baseline	29.62(28.38 to 30.87)	29.03(27.8 to 30.26)	
12 weeks	30.59(29.38 to 31.8)	29.34(28.15 to 30.54)	0.48^a^

a Time to treatment interaction according to two-way repeated measure ANOVA. Data are not conforming to a normal distribution were log and square root transformed.

## Discussion

There is not much scientific literature on the commercially available pine bark extract (oligopin or pycnogenol). To the best of our knowledge, this is the first randomized, double-blind, placebo-controlled trial of oligopin supplementation in the women with PCOS. We hypothesize that including oligopin in our subjects would induce benefits; however, with the exception of change in the FSH level, our intervention failed to change the levels of androgens and metabolic profile. These results were not accompanied by any unexpected safety finding.

This trial showed that an antioxidant intervention based on the oligopin supplementation had no effect on the serum androgen levels, except for a small increase in the FSH level. The changes in the FSH levels observed in this trial over the 3-month period were difficult to explain, even though the mean FSH was increased following oligopin treatment; there were no significant changes in insulin or androgen levels. On the other hand, although the mean FSH levels were lower for the women with PCOS, as compared to those with normal ovaries (28), this small rise in the FSH concentrations was not clinically important in the patients with PCOS. Whether the effect FSH on raising oligopin was partly due to catechin is unclear. It has been reported that there is a significant dose-response relationship between catechein supplementation and FSH levels in the PCOS rats (29).

No differences could be demonstrated in any of metabolic profiles and anthropometrics parameters, blood pressure and hs-CRP levels, except for the increase in the ALP levels in the placebo group, as compared to the oligopin one. Our findings showed that oligopin failed to significantly influence cardiovascular disease risk factors (insulin, fasting glucose, lipid profiles and hs-CRP); this is an observation previously recorded in the case of overweight and obese adults (30). The meta-analysis by Malekamadi et al. and collaborators also showed several biological effects of this extract, such as decreased glycemia and lipid profile, reduced weight and blood pressure and declined hs-CRP level, which could be due to pooling low quality and high heterogeneity studies (16). Notably, the meta-analysis of randomized trials indicated that pycnogenol significantly raised the AST levels and decreased the Gamma–Glutamyl Transferase (GGT) concentration by 1.53 U/I (16). In the present study, we did not observe any significant changes in the ALT and AST levels in the oligopin group. However, there was an increase in the ALP levels in both treatment groups, which was greater in the placebo group, as compared to oligopin. Overall, based on the results and the previous study, oligopin did not show any toxic effects on the liver function (16). The hypoglycemic effects of the pine bark extract may be related to the inhibition of the alpha glycosidase activity in the small intestinal brush border due to procyanidins (flavonoids), independent of the effect on insulin secretion (19, 31). We speculate that the efficiency of oligopin on glycemia and HbA1c depends on the baseline glycemia. We enrolled the patients with normal glycemia (glycated hemoglobin levels less than 6% and FBS less than 126 mg/dl).

Pycnogenol (the pine bark extract) has been explored as a potential natural antihypertensive agent. While the results have not been consistent, pycnogenol supplementation has been shown to reduce the systolic and diastolic blood pressure (32). The effect is mediated *via* nitric oxide (NO) production (32) or angiotensin converting enzyme (ACE) inhibition (33) and /or reduction of endothelin -1 (17). In our study, blood pressure was reduced after oligopin treatment for 3 months, although the difference was not significant. As most of our study participants displayed well-controlled blood pressure levels (97.5% SBP<140 mmHg, 85% DBS <90 mmHg) at the baseline, we postulate that oligopin supplementation could exert favorable effects on blood pressure only among hypertensive patients (16, 33, 34). On the other hand, the subgroup analysis in the recent meta-analysis indicated that the effect of this extract on blood pressure was more prominent in the trails with a longer intervention duration (>12 weeks) (32). As a result, a longer period is required to obtain results.

Supplementation with oligopin has been suggested to decrease the levels of CRP, with an anti-inflammatory effect (35). However, our data failed to show any significant change in the hs-CRP levels with oligopin administration. We have not measured the circulation of other inflammatory factors in this study; so, we cannot conclude that oligopin is ineffective on inflammation.

There are some possible explanations for the apparent lack of a positive finding in our study. First, the dose of oligopin might have been inadequate. One study involved the use of oligopin in type 2 diabetes, showing that a daily consumption in the amount of 100 mg/d to 200 mg/d was required to achieve a protective effect (36). It is uncertain whether the dose required to achieve androgen reduction is similar, or the response to oligopin may be different in different subjects; we included the PCOS women. Moreover, the recent meta-analysis suggested a possible benefit for pine bark extract supplementation when adding other treatments and no benefit when consuming pine bark extracts as a solitary therapy (32).

Furthermore, the impact of oligopin on hormonal and cadiometabolic profiles may be different for the two sexes; sex may be a modifier of the effect of the pine bark extract on the cardiometabolic profile, or the link between oligopin and PCOS may be appreciated only in the sub-population of the PCOS patients; so, insulin resistance vs. noninsulin resistance and also, between lean and obese should be noted. This can be partially explained by the fact that a large number of cases in the population in our trial were not insulin-resistant (mean HOMA <3.8) and they were relatively lean (overweight on average).

## Conclusion

This study demonstrated, for the first time, that a nutraceutical intervention based on a 3-month 50 mg oligopin (pine bark extract) administration among PCOS women was safe, but it did not improve androgen or metabolic/anthropometric parameters.

## Data Availability Statement

The raw data supporting the conclusions of this article will be made available by the authors, without undue reservation.

## Ethics Statement

The studies involving human participants were reviewed and approved by Ethics approval was obtained from the Ethics Committee of Endocrinology Metabolism Research Institute, Tehran University of Medical Sciences (REC.1396.00163). The patients/participants provided their written informed consent to participate in this study.

## Author Contributions

AM: study conception and design, acquisition of data, analysis and interpretation of data, drafting of manuscript, critical revision. MiS: acquisition of data; MM-T: critical revision; AM-G: critical revision; SK: acquisition of data; HG: acquisition of data, NS: critical revision; MaS: acquisition of data; FB: critical revision; MQ: analysis and interpretation of data; SH: study conception and design. All authors contributed to the article and approved the submitted version.

## Funding

This study was supported by the Alborz University of Medical Sciences, Karaj, Iran and by Endocrinology and Metabolism Research Institute, Tehran University of Medical Sciences, Tehran, Iran. The funders had no role in study design, data collection and analysis, decision to publish, or preparation of the manuscript.

## Conflict of Interest

The authors declare that the research was conducted in the absence of any commercial or financial relationships that could be construed as a potential conflict of interest.

## Abbreviations

ALT, Alanine Transaminase; ALP, Alkaline Phosphatase; AST, Aspartate Transaminase; BUN, Blood Urea Nitrogen; BIA, Body Impedance Analyzer; BMI, Body Mass Index; Cr, Creatinine; DHEA, Dehydroepiandrosterone; DBS, Diastolic Blood Pressure; FEM, Fat Free Mass; FM, Fat Mass; *FSH, Follicle-Stimulating* Hormone; FAI, Free Androgen Index; GGT, Gamma–Glutamyl Transferase; HbA1c, Hemoglobin A1c; HDL, High-Density Lipoprotein Cholesterol; hs-CRP, High Sensitive- C Reactive Protein; HOMA, Homeostasis Model Assessment; LH, Increase *Luteinizing Hormone;* LDL, Low-Density Lipoprotein Cholesterol; NO, Nitric Oxide; PCOM, Polycystic Ovarian Morphology; *PCOS, Polycystic Ovary Syndrome*; SHBG, Sex Hormone Binding Globulin; SBP, Systolic Blood Pressure; TSH, Thyroid Stimulating Hormone; TC, Total Cholesterol; TG, Triglycerides.
